# 16-Isopropyl-5,9-dimethyl­tetra­cyclo­[10.2.2.0^1,10^.0^4,9^]hexa­dec-15-ene-5,14-dimethanol

**DOI:** 10.1107/S1600536811000705

**Published:** 2011-01-12

**Authors:** Jian Li, Xiao-ping Rao, Shi-bin Shang, Yanqing Gao

**Affiliations:** a Institute of Chemical Industry of Forest Products, Chinese Academy of Forestry, Nanjing, 210042, People’s Republic of China

## Abstract

The title compound, C_23_H_38_O_2_, a tetra­cyclo­[10.2.2.0^1,10^.0^4,9^] hexa­decane structure, crystallized with four independent mol­ecules in the asymmetric unit. In the crystal, these independent mol­ecules are linked by O—H⋯O hydrogen bonds, forming a polymeric chain propagating in [100]

## Related literature

For the isolation of acrylic modified rosin, see: Aldrich (1971 ▶). For the crystal structure of 16-isopropyl-5,9-dimethyl­tetra­cyclo­[10.2.2.01,10.04,9]hexa­dec-15-ene-5,14-dicarb­oxy­lic acid, see: Wang *et al.* (2009 ▶). 
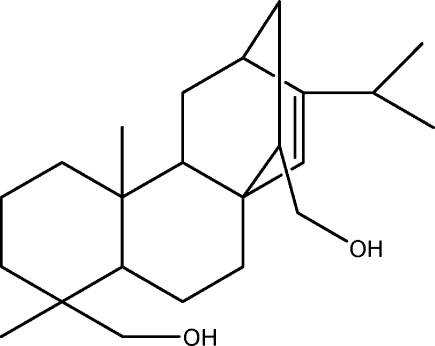


## Experimental

### 

#### Crystal data

C_23_H_38_O_2_
*M* *_r_* = 346.53
Monoclinic, 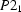

*a* = 12.986 (3) Å
*b* = 25.583 (5) Å
*c* = 14.292 (3) Å
β = 116.19 (3)°
*V* = 4260.6 (19) Å^3^
*Z* = 8
Mo *K*α radiation
μ = 0.07 mm^−1^
*T* = 293 K
0.30 × 0.20 × 0.10 mm

#### Data collection

Enraf–Nonius CAD-4 diffractometer
Absorption correction: ψ scan (North *et al.* 1968 ▶) *T*_min_ = 0.980, *T*_max_ = 0.993
8392 measured reflections
8024 independent reflections
4643 reflections with *I* > 2σ(*I*)
*R*_int_ = 0.028
3 standard reflections every 200 reflections  intensity decay: 1%

#### Refinement

*R*[*F* ^2^ > 2σ(*F* ^2^)] = 0.056
*wR*(*F* ^2^) = 0.160
*S* = 1.00
8024 reflections
917 parameters
13 restraints
H-atom parameters constrained
Δρ_max_ = 0.30 e Å^−3^
Δρ_min_ = −0.14 e Å^−3^

### 

Data collection: *CAD-4 EXPRESS* (Enraf–Nonius, 1994) ▶; cell refinement: *CAD-4 EXPRESS* ▶; data reduction: *XCAD4* (Harms & Wocadlo,1995 ▶); program(s) used to solve structure: *SHELXS97* (Sheldrick, 2008 ▶); program(s) used to refine structure: *SHELXL97* (Sheldrick, 2008 ▶); molecular graphics: *SHELXTL* (Sheldrick, 2008 ▶); software used to prepare material for publication: *SHELXTL*.

## Supplementary Material

Crystal structure: contains datablocks I, global. DOI: 10.1107/S1600536811000705/su2242sup1.cif

Structure factors: contains datablocks I. DOI: 10.1107/S1600536811000705/su2242Isup2.hkl

Additional supplementary materials:  crystallographic information; 3D view; checkCIF report

## Figures and Tables

**Table 1:** Hydrogen-bond geometry (Å, °)

*D*—H⋯*A*	*D*—H	H⋯*A*	*D*⋯*A*	*D*—H⋯*A*
O1—H1*O*⋯O6^i^	0.82	1.99	2.717 (6)	148
O2—H2*O*⋯O7^ii^	0.85	1.84	2.692 (5)	179
O3—H3*O*⋯O2^iii^	0.82	1.92	2.712 (5)	163
O4—H4*O*⋯O5^iv^	0.82	2.11	2.694 (5)	128
O5—H5*O*⋯O8^v^	0.82	1.92	2.682 (5)	155
O6—H6*O*⋯O3^vi^	0.82	2.03	2.704 (6)	139
O7—H7*O*⋯O4^vii^	0.82	2.02	2.692 (5)	139
O8—H8*O*⋯O1^viii^	0.82	1.87	2.677 (6)	166

Symmetry codes: (i) 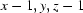; (ii) 
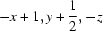
; (iii) 
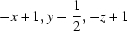
; (iv) 
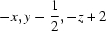
; (v) 
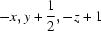
; (vi) 
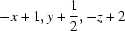
; (vii) 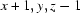; (viii) 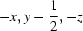.

**Fig. 1.**
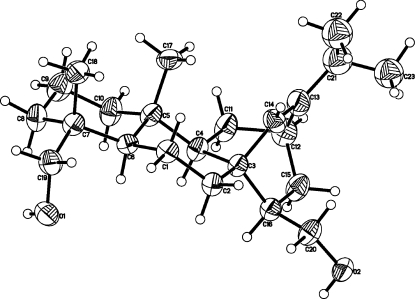
A view of the molecular structure of one of the four independent molecules of the title compound. The displacement ellipsoids are drawn at the 30% probability level.

**Fig. 2.**
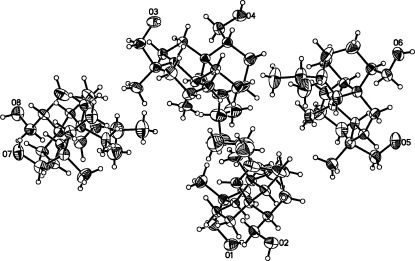
A view of the four independent molecules of the title compound. The displacement ellipsoids are drawn at the 30% probability level.

**Fig. 3.**
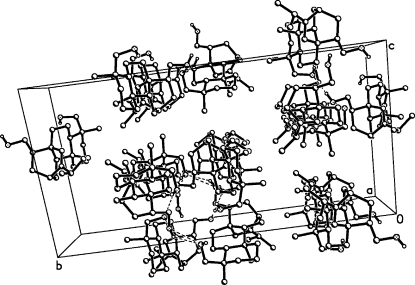
A perspective view along the a-axis of the crystal packing of the title compound. The O-H···O hydrogen bonds are shown as dashed lines (see Table 1 for details).

## supplementary crystallographic information

### Comment

16-Isopropyl-5,9-dimethyltetracyclo[10.2.2.0^1,10^.0^4,9^]hexadec-15-ene-5,14- dicarboxylic acid was isolated from acrylic modified rosin (Aldrich, 1971), and its crystal structure has been reported previously (Wang *et al.*, 2009). The title compound was obtained on reduction of this dicarboxylic acid.

The molecular structure of one of the independent molecules of the title compound is shown in Fig. 1. The asymmetric unit consists of four crystallographically independent molecules (Fig. 2).

In the crystal the independent molecules are linked via O–H···O hydrogen bonds (Table 1), to form a polymer chain propagating in [100], as illustrated in Fig. 3.

### Experimental

16-Isopropyl-5,9-dimethyltetracyclo[10.2.2.0^1,10^.0^4,9^]hexadec-15- ene-5,14-dicarboxylic acid was (20.0 g) dissolved in tetrahydrofuran (300 ml) at 263 K while stirring vigorously, lithium aluminium hydride (8.1 g) was added in batch within 2 h. The reaction was maintained during 1 h at the temperature of reflux, then water (8.1 ml) and 10% sodium hydroxide solution(8.1 ml) were added drop wise at 263 K. The mixture was then filtered and the filtrate was concentrated. The crude product was recrystallized with ethanol, giving the title compound. Crystals, suitable for X-ray diffraction, were obtained by slow evaporation of an ethanol solution.

### Refinement

Only one equivalent of the diffraction data was measured and the absolute structure of the molecules in the crystal are unknown. The structure contains solvent accessible voids of 47 Å^3^ which could possibly be a disordered water molecule. It was not taken into consideration when refining the crystal structure. The C-C bonds of the isopropyl groups wre refined with distance restraints of 1.50 (2) Å. The OH and the C-bound H-atoms were included in calculated positions and treated as riding atoms: O-H = 0.82 - 0.85 Å, C-H = 0.96, 0.9 and 0.98 Å for CH~3~, CH_2_ and CH H-atoms, respectively, with *U*_iso_(H) = k ×*U*_eq_ of the carrier atom, where k = 1.5 for OH and CH_3_ H-atoms and 1.2 for all other H-atoms.

### Crystal data

**Table:**

C_23_H_38_O_2_	*F*(000) = 1536
*M**_r_* = 346.53	*D*_x_ = 1.080 Mg m^−^^3^
Monoclinic, *P*2_1_	Mo *K*α radiation, λ = 0.71073 Å
Hall symbol: P 2yb	Cell parameters from 25 reflections
*a* = 12.986 (3) Å	θ = 9–13°
*b* = 25.583 (5) Å	µ = 0.07 mm^−^^1^
*c* = 14.292 (3) Å	*T* = 293 K
β = 116.19 (3)°	Rod, colourless
*V* = 4260.6 (19) Å^3^	0.30 × 0.20 × 0.10 mm
*Z* = 8	

### Data collection

**Table:**

Enraf–Nonius CAD-4 diffractometer	4643 reflections with *I* > 2σ(*I*)
Radiation source: fine-focus sealed tube	*R*_int_ = 0.028
graphite	θ_max_ = 25.4°, θ_min_ = 1.6°
ω/2θ scans	*h* = 0→15
Absorption correction: ψ scan (North *et al.* 1968)	*k* = 0→30
*T*_min_ = 0.980, *T*_max_ = 0.993	*l* = −17→15
8392 measured reflections	3 standard reflections every 200 reflections
8024 independent reflections	intensity decay: 1%

### Refinement

**Table:**

Refinement on *F*^2^	Primary atom site location: structure-invariant direct methods
Least-squares matrix: full	Secondary atom site location: difference Fourier map
*R*[*F*^2^ > 2σ(*F*^2^)] = 0.056	Hydrogen site location: inferred from neighbouring sites
*wR*(*F*^2^) = 0.160	H-atom parameters constrained
*S* = 1.00	*w* = 1/[σ^2^(*F*_o_^2^) + (0.0716*P*)^2^ + 1.*P*] where *P* = (*F*_o_^2^ + 2*F*_c_^2^)/3
8024 reflections	(Δ/σ)_max_ < 0.001
917 parameters	Δρ_max_ = 0.30 e Å^−^^3^
13 restraints	Δρ_min_ = −0.14 e Å^−^^3^

### Special details

**Table:**

Geometry. All esds (except the esd in the dihedral angle between two l.s. planes) are estimated using the full covariance matrix. The cell esds are taken into account individually in the estimation of esds in distances, angles and torsion angles; correlations between esds in cell parameters are only used when they are defined by crystal symmetry. An approximate (isotropic) treatment of cell esds is used for estimating esds involving l.s. planes.
Refinement. Refinement of F^2^ against ALL reflections. The weighted R-factor wR and goodness of fit S are based on F^2^, conventional R-factors R are based on F, with F set to zero for negative F^2^. The threshold expression of F^2^ > 2sigma(F^2^) is used only for calculating R-factors(gt) etc. and is not relevant to the choice of reflections for refinement. R-factors based on F^2^ are statistically about twice as large as those based on F, and R- factors based on ALL data will be even larger.

### Fractional atomic coordinates and isotropic or equivalent isotropic displacement parameters (Å^2^)

**Table:**

	*x*	*y*	*z*	*U*_iso_*/*U*_eq_	
O1	−0.2354 (3)	0.50237 (16)	0.1961 (3)	0.0794 (12)	
H1O	−0.2662	0.5096	0.2336	0.119*	
O2	0.3722 (3)	0.62999 (15)	0.3156 (3)	0.0725 (11)	
H2O	0.3296	0.6456	0.2591	0.109*	
C1	0.0449 (4)	0.4625 (2)	0.2538 (4)	0.0535 (13)	
H1A	0.0856	0.4296	0.2643	0.064*	
H1B	−0.0179	0.4623	0.1841	0.064*	
C2	0.1255 (4)	0.5069 (2)	0.2632 (4)	0.0558 (13)	
H2A	0.0815	0.5391	0.2420	0.067*	
H2B	0.1587	0.5007	0.2153	0.067*	
C3	0.2223 (4)	0.5144 (2)	0.3723 (3)	0.0488 (12)	
C4	0.1786 (4)	0.5106 (2)	0.4566 (4)	0.0550 (13)	
H4	0.1335	0.5424	0.4487	0.066*	
C5	0.0951 (4)	0.4644 (2)	0.4462 (4)	0.0558 (13)	
C6	−0.0028 (4)	0.4674 (2)	0.3328 (3)	0.0474 (12)	
H6	−0.0307	0.5035	0.3254	0.057*	
C7	−0.1106 (4)	0.4331 (2)	0.3097 (4)	0.0591 (14)	
C8	−0.1499 (5)	0.4414 (3)	0.3969 (4)	0.0708 (16)	
H8A	−0.1823	0.4762	0.3900	0.085*	
H8B	−0.2100	0.4164	0.3872	0.085*	
C9	−0.0556 (5)	0.4353 (3)	0.5040 (5)	0.086 (2)	
H9A	−0.0277	0.3996	0.5137	0.103*	
H9B	−0.0849	0.4422	0.5545	0.103*	
C10	0.0446 (5)	0.4731 (3)	0.5230 (4)	0.0744 (18)	
H10A	0.0173	0.5089	0.5167	0.089*	
H10B	0.1043	0.4683	0.5934	0.089*	
C11	0.2823 (4)	0.5151 (3)	0.5664 (4)	0.0736 (17)	
H11A	0.2702	0.5437	0.6049	0.088*	
H11B	0.2901	0.4830	0.6053	0.088*	
C12	0.3907 (5)	0.5247 (3)	0.5528 (4)	0.0805 (19)	
H12	0.4573	0.5270	0.6212	0.097*	
C13	0.4067 (5)	0.4803 (3)	0.4906 (5)	0.087 (2)	
C14	0.3182 (5)	0.4761 (2)	0.3976 (4)	0.0646 (15)	
H14	0.3156	0.4506	0.3502	0.078*	
C15	0.3774 (5)	0.5744 (2)	0.4934 (4)	0.0718 (16)	
H15A	0.4474	0.5816	0.4870	0.086*	
H15B	0.3641	0.6032	0.5309	0.086*	
C16	0.2773 (4)	0.5701 (2)	0.3855 (4)	0.0544 (13)	
H16	0.2194	0.5961	0.3798	0.065*	
C17	0.1589 (5)	0.4119 (2)	0.4722 (5)	0.0772 (18)	
H17A	0.2261	0.4147	0.5377	0.116*	
H17B	0.1810	0.4031	0.4184	0.116*	
H17C	0.1094	0.3852	0.4768	0.116*	
C18	−0.0901 (5)	0.3747 (2)	0.3006 (5)	0.0719 (16)	
H18A	−0.1600	0.3558	0.2832	0.108*	
H18B	−0.0318	0.3623	0.3658	0.108*	
H18C	−0.0656	0.3696	0.2469	0.108*	
C19	−0.2071 (5)	0.4487 (2)	0.2061 (4)	0.0702 (16)	
H19A	−0.1861	0.4390	0.1512	0.084*	
H19B	−0.2750	0.4287	0.1956	0.084*	
C20	0.3157 (5)	0.5811 (2)	0.3008 (4)	0.0657 (15)	
H20A	0.3672	0.5535	0.3011	0.079*	
H20B	0.2493	0.5808	0.2333	0.079*	
C21	0.5125 (7)	0.4473 (4)	0.5347 (7)	0.119 (3)	
H21	0.5327	0.4351	0.6057	0.143*	
C22	0.4941 (10)	0.4023 (4)	0.4569 (9)	0.174 (5)	
H22A	0.5640	0.3828	0.4785	0.261*	
H22B	0.4726	0.4165	0.3886	0.261*	
H22C	0.4343	0.3797	0.4552	0.261*	
C23	0.6059 (8)	0.4670 (4)	0.5227 (8)	0.143 (3)	
H23A	0.6638	0.4405	0.5407	0.214*	
H23B	0.6367	0.4967	0.5675	0.214*	
H23C	0.5815	0.4774	0.4515	0.214*	
O3	0.4090 (3)	0.10639 (17)	0.6457 (3)	0.0779 (12)	
H3O	0.4714	0.1197	0.6590	0.117*	
O4	−0.0145 (3)	0.16146 (16)	0.9101 (3)	0.0739 (11)	
H4O	0.0459	0.1515	0.9577	0.111*	
C24	0.2672 (5)	0.2709 (2)	0.6054 (5)	0.0643 (15)	
H24A	0.2387	0.3064	0.5997	0.077*	
H24B	0.3261	0.2660	0.6761	0.077*	
C25	0.3206 (5)	0.2640 (2)	0.5303 (5)	0.0752 (17)	
H25A	0.2635	0.2709	0.4595	0.090*	
H25B	0.3830	0.2888	0.5473	0.090*	
C26	0.3656 (5)	0.2090 (2)	0.5377 (5)	0.0750 (17)	
H26A	0.4254	0.2032	0.6078	0.090*	
H26B	0.4000	0.2054	0.4900	0.090*	
C27	0.2746 (4)	0.1671 (2)	0.5129 (4)	0.0567 (13)	
C28	0.2137 (4)	0.17624 (18)	0.5840 (4)	0.0471 (11)	
H28	0.2744	0.1712	0.6549	0.057*	
C29	0.1685 (4)	0.2326 (2)	0.5847 (4)	0.0540 (13)	
C30	0.1232 (4)	0.1361 (2)	0.5736 (4)	0.0520 (12)	
H30A	0.1516	0.1013	0.5710	0.062*	
H30B	0.0547	0.1419	0.5090	0.062*	
C31	0.0928 (5)	0.13969 (19)	0.6660 (4)	0.0544 (13)	
H31A	0.0323	0.1148	0.6552	0.065*	
H31B	0.1596	0.1296	0.7291	0.065*	
C32	0.0538 (4)	0.19368 (19)	0.6822 (4)	0.0462 (11)	
C33	0.1373 (4)	0.23639 (19)	0.6781 (4)	0.0514 (13)	
H33	0.2094	0.2317	0.7413	0.062*	
C34	0.0910 (5)	0.2907 (2)	0.6884 (5)	0.0717 (16)	
H34A	0.0725	0.3109	0.6255	0.086*	
H34B	0.1491	0.3095	0.7468	0.086*	
C35	−0.0179 (6)	0.2836 (2)	0.7056 (6)	0.0786 (19)	
H35	−0.0487	0.3177	0.7119	0.094*	
C36	0.0191 (6)	0.2521 (2)	0.8076 (5)	0.0739 (17)	
H36A	0.0812	0.2701	0.8643	0.089*	
H36B	−0.0448	0.2491	0.8251	0.089*	
C37	0.0583 (4)	0.1983 (2)	0.7935 (4)	0.0504 (12)	
H37	0.1386	0.1943	0.8449	0.060*	
C38	−0.1049 (5)	0.2539 (3)	0.6168 (5)	0.0789 (19)	
C39	−0.0663 (4)	0.2068 (3)	0.6080 (4)	0.0636 (15)	
H39	−0.1125	0.1831	0.5576	0.076*	
C40	0.0667 (5)	0.2474 (2)	0.4809 (4)	0.0717 (17)	
H40A	0.0941	0.2669	0.4390	0.108*	
H40B	0.0134	0.2683	0.4943	0.108*	
H40C	0.0291	0.2162	0.4445	0.108*	
C41	0.1937 (5)	0.1642 (3)	0.3966 (4)	0.0814 (19)	
H41A	0.2362	0.1539	0.3591	0.122*	
H41B	0.1595	0.1978	0.3725	0.122*	
H41C	0.1346	0.1389	0.3851	0.122*	
C42	0.3345 (5)	0.1133 (2)	0.5388 (5)	0.0676 (16)	
H42A	0.2763	0.0862	0.5170	0.081*	
H42B	0.3780	0.1091	0.4989	0.081*	
C43	−0.0092 (5)	0.1550 (2)	0.8113 (4)	0.0629 (15)	
H43A	−0.0864	0.1547	0.7550	0.075*	
H43B	0.0263	0.1217	0.8109	0.075*	
C44	−0.2276 (6)	0.2714 (3)	0.5497 (6)	0.098 (2)	
H44A	−0.2617	0.2421	0.5019	0.118*	
C45	−0.2956 (6)	0.2751 (4)	0.6056 (6)	0.113 (3)	
H45A	−0.3708	0.2878	0.5597	0.169*	
H45B	−0.2599	0.2989	0.6630	0.169*	
H45C	−0.3019	0.2413	0.6315	0.169*	
C46	−0.2399 (7)	0.3144 (3)	0.4825 (6)	0.114 (3)	
H46A	−0.2057	0.3059	0.4371	0.170*	
H46B	−0.2025	0.3445	0.5235	0.170*	
H46C	−0.3200	0.3218	0.4416	0.170*	
O5	−0.0878 (3)	0.57521 (17)	0.9800 (3)	0.0851 (13)	
H5O	−0.1350	0.5591	0.9923	0.128*	
O6	0.5896 (3)	0.51875 (17)	1.2480 (3)	0.0789 (12)	
H6O	0.5805	0.5334	1.2949	0.118*	
C47	0.0097 (5)	0.4123 (2)	0.9244 (5)	0.0778 (18)	
H47A	0.0174	0.4150	0.9949	0.093*	
H47B	0.0322	0.3772	0.9155	0.093*	
C48	−0.1160 (5)	0.4204 (3)	0.8479 (6)	0.088 (2)	
H48A	−0.1261	0.4153	0.7771	0.106*	
H48B	−0.1634	0.3951	0.8614	0.106*	
C49	−0.1524 (5)	0.4747 (3)	0.8597 (5)	0.0824 (19)	
H49A	−0.1477	0.4783	0.9290	0.099*	
H49B	−0.2320	0.4795	0.8098	0.099*	
C50	−0.0809 (5)	0.5173 (2)	0.8434 (4)	0.0605 (14)	
C51	0.0462 (4)	0.50645 (19)	0.9154 (4)	0.0556 (13)	
H51	0.0531	0.5100	0.9862	0.067*	
C52	0.0913 (5)	0.4509 (2)	0.9117 (4)	0.0595 (14)	
C53	0.2112 (4)	0.4457 (2)	1.0075 (4)	0.0559 (13)	
H53	0.1974	0.4494	1.0693	0.067*	
C54	0.3000 (4)	0.4887 (2)	1.0170 (4)	0.0520 (13)	
C55	0.2476 (4)	0.5429 (2)	1.0013 (4)	0.0565 (13)	
H55A	0.2992	0.5674	0.9920	0.068*	
H55B	0.2411	0.5528	1.0640	0.068*	
C56	0.1297 (4)	0.5474 (2)	0.9085 (4)	0.0558 (13)	
H56A	0.1369	0.5423	0.8444	0.067*	
H56B	0.0992	0.5822	0.9070	0.067*	
C57	0.2701 (5)	0.3919 (2)	1.0186 (5)	0.0782 (18)	
H57A	0.2644	0.3723	1.0742	0.094*	
H57B	0.2304	0.3723	0.9544	0.094*	
C58	0.3940 (5)	0.3974 (3)	1.0419 (6)	0.088 (2)	
H58	0.4300	0.3631	1.0487	0.105*	
C59	0.4041 (5)	0.4290 (3)	0.9578 (6)	0.086 (2)	
C60	0.3523 (5)	0.4776 (3)	0.9426 (4)	0.0679 (16)	
H60	0.3498	0.5011	0.8919	0.081*	
C61	0.4532 (6)	0.4282 (3)	1.1390 (6)	0.098 (2)	
H61A	0.5340	0.4311	1.1560	0.117*	
H61B	0.4468	0.4102	1.1960	0.117*	
C62	0.4005 (5)	0.4835 (2)	1.1264 (4)	0.0636 (14)	
H62	0.3685	0.4865	1.1768	0.076*	
C63	−0.1150 (5)	0.5702 (3)	0.8732 (5)	0.0792 (19)	
H63A	−0.0767	0.5979	0.8543	0.095*	
H63B	−0.1970	0.5751	0.8326	0.095*	
C64	−0.1094 (5)	0.5238 (3)	0.7281 (4)	0.0779 (18)	
H64A	−0.1015	0.4908	0.7001	0.117*	
H64B	−0.0578	0.5487	0.7213	0.117*	
H64C	−0.1869	0.5361	0.6906	0.117*	
C65	0.0978 (5)	0.4385 (3)	0.8078 (5)	0.0758 (17)	
H65A	0.0219	0.4388	0.7513	0.114*	
H65B	0.1316	0.4046	0.8125	0.114*	
H65C	0.1439	0.4644	0.7956	0.114*	
C66	0.4910 (5)	0.5260 (2)	1.1525 (4)	0.0645 (15)	
H66A	0.5145	0.5277	1.0969	0.077*	
H66B	0.4566	0.5594	1.1547	0.077*	
C67	0.4671 (6)	0.4063 (3)	0.9017 (6)	0.102 (3)	
H67	0.5246	0.3847	0.9568	0.123*	
C68	0.5382 (8)	0.4397 (4)	0.8765 (8)	0.129 (3)	
H68A	0.5810	0.4193	0.8493	0.194*	
H68B	0.5904	0.4578	0.9381	0.194*	
H68C	0.4915	0.4646	0.8251	0.194*	
C69	0.4039 (7)	0.3658 (4)	0.8217 (7)	0.133 (3)	
H69A	0.4544	0.3507	0.7962	0.199*	
H69B	0.3398	0.3817	0.7648	0.199*	
H69C	0.3769	0.3390	0.8523	0.199*	
O7	0.7637 (3)	0.17940 (18)	−0.1371 (3)	0.0820 (13)	
H7O	0.8280	0.1695	−0.0964	0.123*	
O8	0.2650 (3)	0.05258 (16)	−0.0216 (3)	0.0788 (12)	
H8O	0.2461	0.0354	−0.0751	0.118*	
C70	0.8051 (4)	0.1997 (3)	0.1952 (4)	0.0710 (16)	
H70A	0.8147	0.2027	0.2663	0.085*	
H70B	0.8214	0.1638	0.1845	0.085*	
C71	0.8912 (5)	0.2354 (3)	0.1816 (5)	0.085 (2)	
H71A	0.8787	0.2712	0.1965	0.101*	
H71B	0.9684	0.2254	0.2304	0.101*	
C72	0.8786 (4)	0.2318 (3)	0.0714 (4)	0.0713 (16)	
H72A	0.8977	0.1965	0.0594	0.086*	
H72B	0.9332	0.2554	0.0643	0.086*	
C73	0.7580 (4)	0.2452 (2)	−0.0120 (4)	0.0582 (14)	
C74	0.6696 (4)	0.2130 (2)	0.0078 (4)	0.0527 (13)	
H74	0.6863	0.1769	−0.0036	0.063*	
C75	0.6798 (4)	0.2121 (2)	0.1198 (4)	0.0523 (13)	
C76	0.5455 (4)	0.2203 (2)	−0.0702 (4)	0.0589 (14)	
H76A	0.5403	0.2228	−0.1398	0.071*	
H76B	0.5165	0.2526	−0.0553	0.071*	
C77	0.4717 (4)	0.1744 (2)	−0.0656 (4)	0.0578 (14)	
H77A	0.3926	0.1807	−0.1154	0.069*	
H77B	0.4970	0.1428	−0.0865	0.069*	
C78	0.4766 (4)	0.1659 (2)	0.0420 (4)	0.0521 (12)	
C79	0.6066 (4)	0.1668 (2)	0.1293 (3)	0.0512 (12)	
H79	0.6419	0.1346	0.1201	0.061*	
C80	0.6051 (5)	0.1621 (3)	0.2361 (4)	0.0757 (17)	
H80A	0.6544	0.1334	0.2747	0.091*	
H80B	0.6355	0.1939	0.2755	0.091*	
C81	0.4834 (5)	0.1526 (3)	0.2245 (4)	0.0773 (19)	
H81A	0.4845	0.1493	0.2932	0.093*	
C82	0.4364 (6)	0.1032 (3)	0.1615 (5)	0.087 (2)	
H82A	0.3601	0.0961	0.1549	0.105*	
H82B	0.4854	0.0738	0.1966	0.105*	
C83	0.4315 (4)	0.1105 (2)	0.0500 (4)	0.0571 (13)	
H83A	0.4815	0.0843	0.0410	0.069*	
C84	0.4093 (5)	0.2043 (2)	0.0715 (5)	0.0668 (15)	
H84	0.3689	0.2315	0.0275	0.080*	
C85	0.4102 (5)	0.1972 (3)	0.1668 (5)	0.0786 (19)	
C86	0.7501 (5)	0.2334 (3)	−0.1170 (5)	0.0762 (18)	
H86A	0.6759	0.2452	−0.1691	0.091*	
H86B	0.8083	0.2536	−0.1259	0.091*	
C87	0.7344 (6)	0.3050 (2)	−0.0182 (6)	0.087 (2)	
H87A	0.6569	0.3118	−0.0686	0.131*	
H87B	0.7865	0.3227	−0.0386	0.131*	
H87C	0.7453	0.3176	0.0489	0.131*	
C88	0.6460 (5)	0.2652 (2)	0.1501 (5)	0.0748 (18)	
H88A	0.5711	0.2751	0.0985	0.112*	
H88B	0.7007	0.2913	0.1540	0.112*	
H88C	0.6452	0.2621	0.2167	0.112*	
C89	0.3108 (5)	0.1022 (2)	−0.0334 (4)	0.0671 (15)	
H89A	0.2620	0.1302	−0.0299	0.081*	
H89B	0.3104	0.1037	−0.1014	0.081*	
C90	0.3458 (7)	0.2273 (3)	0.2128 (7)	0.109 (3)	
H90	0.3306	0.2017	0.2562	0.131*	
C91	0.2334 (7)	0.2459 (4)	0.1414 (7)	0.121 (3)	
H91A	0.1972	0.2618	0.1800	0.182*	
H91B	0.1876	0.2170	0.1018	0.182*	
H91C	0.2406	0.2711	0.0950	0.182*	
C92	0.4157 (7)	0.2687 (4)	0.2882 (6)	0.117 (3)	
H92A	0.3705	0.2851	0.3179	0.176*	
H92B	0.4389	0.2944	0.2524	0.176*	
H92C	0.4824	0.2530	0.3426	0.176*	

### Atomic displacement parameters (Å^2^)

**Table:**

	*U*^11^	*U*^22^	*U*^33^	*U*^12^	*U*^13^	*U*^23^
O1	0.069 (2)	0.080 (3)	0.093 (3)	0.018 (2)	0.040 (2)	0.016 (2)
O2	0.058 (2)	0.073 (3)	0.078 (3)	−0.016 (2)	0.023 (2)	0.014 (2)
C1	0.055 (3)	0.059 (3)	0.042 (3)	−0.007 (3)	0.017 (2)	−0.008 (2)
C2	0.052 (3)	0.069 (4)	0.045 (3)	−0.006 (3)	0.020 (2)	−0.004 (2)
C3	0.047 (3)	0.058 (3)	0.039 (3)	−0.009 (2)	0.017 (2)	−0.002 (2)
C4	0.056 (3)	0.070 (4)	0.038 (3)	−0.009 (3)	0.020 (2)	0.001 (2)
C5	0.057 (3)	0.062 (4)	0.044 (3)	−0.013 (3)	0.018 (3)	−0.001 (2)
C6	0.052 (3)	0.049 (3)	0.037 (3)	−0.001 (2)	0.015 (2)	0.000 (2)
C7	0.056 (3)	0.065 (4)	0.052 (3)	−0.007 (3)	0.020 (3)	−0.001 (3)
C8	0.055 (3)	0.087 (5)	0.072 (4)	−0.008 (3)	0.030 (3)	0.002 (3)
C9	0.077 (4)	0.113 (6)	0.062 (4)	−0.019 (4)	0.026 (3)	0.012 (4)
C10	0.065 (4)	0.110 (5)	0.051 (3)	−0.017 (4)	0.028 (3)	0.001 (3)
C11	0.059 (3)	0.104 (5)	0.045 (3)	−0.028 (3)	0.011 (3)	0.001 (3)
C12	0.063 (4)	0.114 (6)	0.046 (3)	−0.013 (4)	0.007 (3)	0.012 (4)
C13	0.060 (4)	0.119 (6)	0.074 (4)	0.000 (4)	0.021 (4)	0.034 (4)
C14	0.056 (3)	0.073 (4)	0.060 (3)	0.013 (3)	0.020 (3)	0.009 (3)
C15	0.066 (4)	0.083 (4)	0.055 (3)	−0.027 (3)	0.016 (3)	−0.005 (3)
C16	0.054 (3)	0.059 (3)	0.048 (3)	−0.004 (3)	0.020 (3)	−0.001 (2)
C17	0.067 (4)	0.067 (4)	0.080 (4)	0.003 (3)	0.016 (3)	0.029 (3)
C18	0.069 (4)	0.051 (3)	0.091 (4)	−0.010 (3)	0.031 (3)	−0.007 (3)
C19	0.056 (3)	0.075 (4)	0.062 (4)	−0.002 (3)	0.011 (3)	−0.002 (3)
C20	0.075 (4)	0.073 (4)	0.050 (3)	−0.019 (3)	0.028 (3)	−0.005 (3)
C21	0.088 (6)	0.135 (8)	0.130 (7)	0.035 (6)	0.044 (6)	0.034 (6)
C22	0.163 (10)	0.165 (11)	0.154 (10)	0.046 (9)	0.033 (8)	−0.017 (8)
C23	0.114 (7)	0.133 (8)	0.160 (9)	0.006 (6)	0.042 (7)	0.017 (7)
O3	0.057 (2)	0.087 (3)	0.085 (3)	0.019 (2)	0.026 (2)	0.023 (2)
O4	0.077 (3)	0.093 (3)	0.067 (2)	0.018 (2)	0.045 (2)	0.021 (2)
C24	0.073 (4)	0.050 (3)	0.082 (4)	−0.002 (3)	0.045 (3)	0.008 (3)
C25	0.078 (4)	0.068 (4)	0.099 (5)	0.007 (3)	0.057 (4)	0.020 (3)
C26	0.072 (4)	0.078 (4)	0.095 (5)	−0.005 (3)	0.055 (4)	0.004 (4)
C27	0.052 (3)	0.063 (3)	0.058 (3)	0.010 (3)	0.026 (3)	0.009 (3)
C28	0.042 (3)	0.046 (3)	0.046 (3)	0.009 (2)	0.013 (2)	0.005 (2)
C29	0.058 (3)	0.047 (3)	0.061 (3)	0.000 (2)	0.029 (3)	0.003 (2)
C30	0.053 (3)	0.053 (3)	0.051 (3)	−0.007 (2)	0.023 (3)	−0.007 (2)
C31	0.057 (3)	0.048 (3)	0.060 (3)	0.001 (2)	0.027 (3)	0.001 (2)
C32	0.041 (3)	0.047 (3)	0.047 (3)	0.002 (2)	0.016 (2)	0.002 (2)
C33	0.059 (3)	0.040 (3)	0.067 (3)	0.001 (2)	0.039 (3)	0.000 (2)
C34	0.079 (4)	0.061 (4)	0.089 (4)	0.012 (3)	0.049 (4)	0.000 (3)
C35	0.103 (5)	0.061 (4)	0.105 (5)	0.035 (4)	0.076 (5)	0.026 (4)
C36	0.085 (4)	0.071 (4)	0.082 (4)	0.006 (3)	0.052 (4)	−0.001 (3)
C37	0.046 (3)	0.056 (3)	0.046 (3)	0.005 (2)	0.018 (2)	0.003 (2)
C38	0.072 (4)	0.090 (5)	0.086 (5)	0.038 (4)	0.045 (4)	0.037 (4)
C39	0.042 (3)	0.093 (5)	0.052 (3)	0.000 (3)	0.017 (3)	0.003 (3)
C40	0.071 (4)	0.082 (4)	0.060 (3)	0.029 (3)	0.026 (3)	0.029 (3)
C41	0.085 (4)	0.099 (5)	0.055 (3)	0.021 (4)	0.025 (3)	0.018 (3)
C42	0.066 (4)	0.068 (4)	0.072 (4)	0.023 (3)	0.033 (3)	0.011 (3)
C43	0.066 (3)	0.072 (4)	0.062 (3)	−0.015 (3)	0.039 (3)	−0.004 (3)
C44	0.073 (4)	0.125 (7)	0.098 (5)	0.023 (4)	0.040 (4)	0.028 (5)
C45	0.086 (5)	0.130 (7)	0.126 (7)	0.023 (5)	0.051 (5)	0.028 (5)
C46	0.092 (5)	0.123 (7)	0.120 (6)	0.018 (5)	0.042 (5)	0.045 (5)
O5	0.074 (3)	0.103 (3)	0.097 (3)	−0.012 (2)	0.055 (3)	−0.038 (3)
O6	0.055 (2)	0.098 (3)	0.061 (2)	0.005 (2)	0.0047 (19)	−0.020 (2)
C47	0.056 (4)	0.072 (4)	0.095 (5)	−0.021 (3)	0.024 (3)	−0.002 (3)
C48	0.061 (4)	0.072 (4)	0.116 (6)	−0.020 (3)	0.025 (4)	−0.016 (4)
C49	0.058 (4)	0.095 (5)	0.092 (5)	−0.002 (4)	0.031 (3)	−0.021 (4)
C50	0.060 (3)	0.062 (3)	0.060 (3)	0.000 (3)	0.026 (3)	−0.007 (3)
C51	0.058 (3)	0.051 (3)	0.063 (3)	−0.004 (3)	0.031 (3)	−0.014 (3)
C52	0.065 (3)	0.050 (3)	0.067 (3)	−0.005 (3)	0.033 (3)	−0.010 (3)
C53	0.056 (3)	0.042 (3)	0.067 (3)	0.001 (2)	0.024 (3)	0.002 (2)
C54	0.052 (3)	0.057 (3)	0.054 (3)	−0.003 (2)	0.030 (3)	−0.003 (2)
C55	0.064 (3)	0.046 (3)	0.064 (3)	0.002 (3)	0.033 (3)	0.002 (3)
C56	0.063 (3)	0.049 (3)	0.055 (3)	0.000 (3)	0.026 (3)	0.001 (2)
C57	0.072 (4)	0.045 (3)	0.098 (5)	0.000 (3)	0.020 (4)	−0.005 (3)
C58	0.064 (4)	0.056 (4)	0.136 (7)	0.003 (3)	0.037 (4)	−0.021 (4)
C59	0.057 (4)	0.084 (5)	0.114 (6)	−0.007 (4)	0.034 (4)	−0.038 (4)
C60	0.068 (4)	0.081 (4)	0.065 (4)	−0.009 (3)	0.039 (3)	−0.020 (3)
C61	0.072 (4)	0.072 (5)	0.120 (6)	0.005 (4)	0.015 (4)	−0.002 (4)
C62	0.056 (3)	0.065 (4)	0.063 (4)	−0.005 (3)	0.020 (3)	−0.005 (3)
C63	0.076 (4)	0.096 (5)	0.075 (4)	0.025 (4)	0.042 (4)	−0.009 (4)
C64	0.080 (4)	0.089 (5)	0.060 (4)	0.015 (4)	0.026 (3)	−0.003 (3)
C65	0.075 (4)	0.078 (4)	0.074 (4)	0.002 (3)	0.032 (3)	−0.023 (3)
C66	0.068 (4)	0.071 (4)	0.056 (3)	−0.011 (3)	0.028 (3)	−0.009 (3)
C67	0.083 (5)	0.107 (6)	0.117 (6)	−0.002 (4)	0.043 (5)	−0.048 (5)
C68	0.153 (8)	0.114 (7)	0.157 (8)	−0.026 (6)	0.102 (7)	−0.031 (6)
C69	0.102 (6)	0.129 (7)	0.166 (8)	−0.018 (5)	0.059 (6)	−0.078 (7)
O7	0.067 (2)	0.103 (4)	0.082 (3)	−0.009 (2)	0.038 (2)	−0.035 (2)
O8	0.081 (3)	0.074 (3)	0.092 (3)	−0.022 (2)	0.048 (3)	−0.012 (2)
C70	0.044 (3)	0.095 (5)	0.058 (3)	0.002 (3)	0.009 (3)	−0.009 (3)
C71	0.038 (3)	0.114 (5)	0.091 (5)	−0.011 (3)	0.019 (3)	−0.032 (4)
C72	0.048 (3)	0.092 (5)	0.072 (4)	−0.001 (3)	0.025 (3)	−0.013 (3)
C73	0.053 (3)	0.063 (4)	0.065 (4)	−0.004 (3)	0.032 (3)	−0.004 (3)
C74	0.040 (3)	0.064 (3)	0.049 (3)	−0.008 (2)	0.015 (2)	−0.007 (2)
C75	0.045 (3)	0.063 (3)	0.047 (3)	−0.002 (2)	0.019 (2)	−0.010 (2)
C76	0.060 (3)	0.066 (4)	0.049 (3)	0.001 (3)	0.022 (3)	0.011 (3)
C77	0.046 (3)	0.075 (4)	0.051 (3)	0.004 (3)	0.019 (2)	0.016 (3)
C78	0.046 (3)	0.057 (3)	0.050 (3)	−0.001 (2)	0.018 (2)	0.000 (2)
C79	0.048 (3)	0.062 (3)	0.039 (3)	0.001 (2)	0.015 (2)	0.002 (2)
C80	0.070 (4)	0.109 (5)	0.048 (3)	−0.003 (4)	0.026 (3)	0.006 (3)
C81	0.070 (4)	0.127 (6)	0.039 (3)	−0.008 (4)	0.028 (3)	0.004 (4)
C82	0.083 (5)	0.116 (6)	0.058 (4)	−0.016 (4)	0.027 (3)	0.021 (4)
C83	0.051 (3)	0.070 (4)	0.045 (3)	−0.004 (3)	0.016 (2)	0.003 (3)
C84	0.054 (3)	0.070 (4)	0.084 (4)	0.007 (3)	0.037 (3)	0.002 (3)
C85	0.066 (4)	0.108 (5)	0.077 (4)	−0.016 (4)	0.044 (4)	−0.029 (4)
C86	0.067 (4)	0.091 (5)	0.084 (4)	−0.006 (3)	0.045 (3)	0.006 (4)
C87	0.098 (5)	0.055 (4)	0.134 (6)	0.000 (3)	0.075 (5)	0.006 (4)
C88	0.075 (4)	0.076 (4)	0.085 (4)	−0.012 (3)	0.046 (4)	−0.037 (3)
C89	0.063 (4)	0.062 (4)	0.063 (3)	−0.015 (3)	0.015 (3)	−0.002 (3)
C90	0.110 (6)	0.118 (7)	0.118 (6)	−0.007 (6)	0.068 (6)	−0.018 (5)
C91	0.099 (6)	0.131 (7)	0.130 (7)	0.026 (5)	0.047 (6)	−0.014 (6)
C92	0.119 (6)	0.127 (7)	0.121 (6)	−0.009 (6)	0.067 (5)	−0.035 (6)

### Geometric parameters (Å, °)

**Table:**

O1—C19	1.413 (7)	O5—C63	1.413 (7)
O1—H1O	0.8200	O5—H5O	0.8200
O2—C20	1.419 (6)	O6—C66	1.412 (6)
O2—H2O	0.8504	O6—H6O	0.8200
C1—C2	1.510 (7)	C47—C52	1.516 (7)
C1—C6	1.513 (6)	C47—C48	1.526 (8)
C1—H1A	0.9700	C47—H47A	0.9700
C1—H1B	0.9700	C47—H47B	0.9700
C2—C3	1.522 (6)	C48—C49	1.499 (9)
C2—H2A	0.9700	C48—H48A	0.9700
C2—H2B	0.9700	C48—H48B	0.9700
C3—C14	1.500 (7)	C49—C50	1.515 (9)
C3—C4	1.545 (6)	C49—H49A	0.9700
C3—C16	1.566 (7)	C49—H49B	0.9700
C4—C11	1.557 (7)	C50—C64	1.529 (7)
C4—C5	1.566 (7)	C50—C51	1.537 (7)
C4—H4	0.9800	C50—C63	1.542 (8)
C5—C10	1.523 (7)	C51—C56	1.541 (7)
C5—C17	1.534 (8)	C51—C52	1.547 (7)
C5—C6	1.559 (6)	C51—H51	0.9800
C6—C7	1.560 (7)	C52—C65	1.557 (7)
C6—H6	0.9800	C52—C53	1.560 (7)
C7—C19	1.510 (7)	C53—C57	1.548 (7)
C7—C18	1.533 (8)	C53—C54	1.557 (7)
C7—C8	1.555 (7)	C53—H53	0.9800
C8—C9	1.487 (8)	C54—C55	1.515 (7)
C8—H8A	0.9700	C54—C60	1.521 (7)
C8—H8B	0.9700	C54—C62	1.538 (7)
C9—C10	1.546 (8)	C55—C56	1.525 (7)
C9—H9A	0.9700	C55—H55A	0.9700
C9—H9B	0.9700	C55—H55B	0.9700
C10—H10A	0.9700	C56—H56A	0.9700
C10—H10B	0.9700	C56—H56B	0.9700
C11—C12	1.522 (8)	C57—C58	1.498 (9)
C11—H11A	0.9700	C57—H57A	0.9700
C11—H11B	0.9700	C57—H57B	0.9700
C12—C15	1.498 (9)	C58—C61	1.481 (9)
C12—C13	1.512 (10)	C58—C59	1.502 (10)
C12—H12	0.9800	C58—H58	0.9800
C13—C14	1.323 (8)	C59—C60	1.384 (9)
C13—C21	1.493 (9)	C59—C67	1.493 (8)
C14—H14	0.9300	C60—H60	0.9300
C15—C16	1.520 (7)	C61—C62	1.547 (8)
C15—H15A	0.9700	C61—H61A	0.9700
C15—H15B	0.9700	C61—H61B	0.9700
C16—C20	1.525 (7)	C62—C66	1.523 (7)
C16—H16	0.9800	C62—H62	0.9800
C17—H17A	0.9600	C63—H63A	0.9700
C17—H17B	0.9600	C63—H63B	0.9700
C17—H17C	0.9600	C64—H64A	0.9600
C18—H18A	0.9600	C64—H64B	0.9600
C18—H18B	0.9600	C64—H64C	0.9600
C18—H18C	0.9600	C65—H65A	0.9600
C19—H19A	0.9700	C65—H65B	0.9600
C19—H19B	0.9700	C65—H65C	0.9600
C20—H20A	0.9700	C66—H66A	0.9700
C20—H20B	0.9700	C66—H66B	0.9700
C21—C23	1.392 (10)	C67—C68	1.417 (9)
C21—C22	1.544 (11)	C67—C69	1.492 (9)
C21—H21	0.9800	C67—H67	0.9800
C22—H22A	0.9600	C68—H68A	0.9600
C22—H22B	0.9600	C68—H68B	0.9600
C22—H22C	0.9600	C68—H68C	0.9600
C23—H23A	0.9600	C69—H69A	0.9600
C23—H23B	0.9600	C69—H69B	0.9600
C23—H23C	0.9600	C69—H69C	0.9600
O3—C42	1.413 (6)	O7—C86	1.437 (7)
O3—H3O	0.8201	O7—H7O	0.8200
O4—C43	1.453 (6)	O8—C89	1.444 (7)
O4—H4O	0.8200	O8—H8O	0.8199
C24—C25	1.524 (8)	C70—C71	1.522 (8)
C24—C29	1.535 (7)	C70—C75	1.537 (7)
C24—H24A	0.9700	C70—H70A	0.9700
C24—H24B	0.9700	C70—H70B	0.9700
C25—C26	1.511 (8)	C71—C72	1.513 (8)
C25—H25A	0.9700	C71—H71A	0.9700
C25—H25B	0.9700	C71—H71B	0.9700
C26—C27	1.516 (8)	C72—C73	1.531 (7)
C26—H26A	0.9700	C72—H72A	0.9700
C26—H26B	0.9700	C72—H72B	0.9700
C27—C41	1.527 (7)	C73—C86	1.489 (8)
C27—C42	1.543 (7)	C73—C74	1.536 (7)
C27—C28	1.555 (7)	C73—C87	1.557 (8)
C28—C30	1.519 (7)	C74—C76	1.513 (7)
C28—C29	1.559 (7)	C74—C75	1.547 (7)
C28—H28	0.9800	C74—H74	0.9800
C29—C40	1.536 (7)	C75—C79	1.542 (7)
C29—C33	1.560 (7)	C75—C88	1.546 (7)
C30—C31	1.539 (7)	C76—C77	1.535 (7)
C30—H30A	0.9700	C76—H76A	0.9700
C30—H30B	0.9700	C76—H76B	0.9700
C31—C32	1.524 (7)	C77—C78	1.526 (6)
C31—H31A	0.9700	C77—H77A	0.9700
C31—H31B	0.9700	C77—H77B	0.9700
C32—C39	1.486 (7)	C78—C84	1.493 (7)
C32—C33	1.558 (7)	C78—C83	1.558 (7)
C32—C37	1.570 (7)	C78—C79	1.597 (6)
C33—C34	1.546 (7)	C79—C80	1.539 (7)
C33—H33	0.9800	C79—H79	0.9800
C34—C35	1.550 (8)	C80—C81	1.534 (8)
C34—H34A	0.9700	C80—H80A	0.9700
C34—H34B	0.9700	C80—H80B	0.9700
C35—C38	1.483 (9)	C81—C85	1.480 (9)
C35—C36	1.545 (8)	C81—C82	1.516 (9)
C35—H35	0.9800	C81—H81A	0.9800
C36—C37	1.513 (7)	C82—C83	1.577 (7)
C36—H36A	0.9700	C82—H82A	0.9700
C36—H36B	0.9700	C82—H82B	0.9700
C37—C43	1.502 (7)	C83—C89	1.508 (7)
C37—H37	0.9800	C83—H83A	0.9800
C38—C39	1.333 (8)	C84—C85	1.370 (8)
C38—C44	1.520 (8)	C84—H84	0.9300
C39—H39	0.9300	C85—C90	1.487 (8)
C40—H40A	0.9600	C86—H86A	0.9700
C40—H40B	0.9600	C86—H86B	0.9700
C40—H40C	0.9600	C87—H87A	0.9600
C41—H41A	0.9600	C87—H87B	0.9600
C41—H41B	0.9600	C87—H87C	0.9600
C41—H41C	0.9600	C88—H88A	0.9600
C42—H42A	0.9700	C88—H88B	0.9600
C42—H42B	0.9700	C88—H88C	0.9600
C43—H43A	0.9700	C89—H89A	0.9700
C43—H43B	0.9700	C89—H89B	0.9700
C44—C46	1.421 (9)	C90—C91	1.443 (9)
C44—C45	1.432 (9)	C90—C92	1.497 (9)
C44—H44A	0.9800	C90—H90	0.9800
C45—H45A	0.9600	C91—H91A	0.9600
C45—H45B	0.9600	C91—H91B	0.9600
C45—H45C	0.9600	C91—H91C	0.9600
C46—H46A	0.9600	C92—H92A	0.9600
C46—H46B	0.9600	C92—H92B	0.9600
C46—H46C	0.9600	C92—H92C	0.9600
			
C19—O1—H1O	109.4	C63—O5—H5O	109.4
C20—O2—H2O	101.3	C66—O6—H6O	109.5
C2—C1—C6	111.5 (4)	C52—C47—C48	114.1 (5)
C2—C1—H1A	109.3	C52—C47—H47A	108.7
C6—C1—H1A	109.3	C48—C47—H47A	108.7
C2—C1—H1B	109.3	C52—C47—H47B	108.7
C6—C1—H1B	109.3	C48—C47—H47B	108.7
H1A—C1—H1B	108.0	H47A—C47—H47B	107.6
C1—C2—C3	114.4 (4)	C49—C48—C47	109.6 (5)
C1—C2—H2A	108.7	C49—C48—H48A	109.7
C3—C2—H2A	108.7	C47—C48—H48A	109.7
C1—C2—H2B	108.7	C49—C48—H48B	109.7
C3—C2—H2B	108.7	C47—C48—H48B	109.7
H2A—C2—H2B	107.6	H48A—C48—H48B	108.2
C14—C3—C2	112.8 (4)	C48—C49—C50	113.8 (5)
C14—C3—C4	108.8 (4)	C48—C49—H49A	108.8
C2—C3—C4	111.7 (4)	C50—C49—H49A	108.8
C14—C3—C16	106.3 (4)	C48—C49—H49B	108.8
C2—C3—C16	111.4 (4)	C50—C49—H49B	108.8
C4—C3—C16	105.6 (4)	H49A—C49—H49B	107.7
C3—C4—C11	109.2 (4)	C49—C50—C64	111.7 (5)
C3—C4—C5	116.8 (4)	C49—C50—C51	108.5 (5)
C11—C4—C5	113.9 (4)	C64—C50—C51	114.8 (4)
C3—C4—H4	105.3	C49—C50—C63	108.6 (5)
C11—C4—H4	105.3	C64—C50—C63	103.9 (5)
C5—C4—H4	105.3	C51—C50—C63	109.2 (4)
C10—C5—C17	108.2 (5)	C50—C51—C56	114.3 (4)
C10—C5—C6	109.2 (4)	C50—C51—C52	117.0 (4)
C17—C5—C6	113.6 (4)	C56—C51—C52	109.6 (4)
C10—C5—C4	108.7 (4)	C50—C51—H51	104.8
C17—C5—C4	111.1 (4)	C56—C51—H51	104.8
C6—C5—C4	105.9 (4)	C52—C51—H51	104.8
C1—C6—C5	110.9 (4)	C47—C52—C51	107.5 (4)
C1—C6—C7	114.8 (4)	C47—C52—C65	108.4 (5)
C5—C6—C7	115.8 (4)	C51—C52—C65	113.6 (5)
C1—C6—H6	104.6	C47—C52—C53	108.6 (5)
C5—C6—H6	104.6	C51—C52—C53	107.2 (4)
C7—C6—H6	104.6	C65—C52—C53	111.5 (5)
C19—C7—C18	105.8 (5)	C57—C53—C54	107.8 (4)
C19—C7—C8	109.0 (5)	C57—C53—C52	115.0 (4)
C18—C7—C8	109.4 (5)	C54—C53—C52	115.5 (4)
C19—C7—C6	109.8 (4)	C57—C53—H53	105.9
C18—C7—C6	113.1 (5)	C54—C53—H53	105.9
C8—C7—C6	109.6 (4)	C52—C53—H53	105.9
C9—C8—C7	113.4 (5)	C55—C54—C60	112.2 (5)
C9—C8—H8A	108.9	C55—C54—C62	110.8 (4)
C7—C8—H8A	108.9	C60—C54—C62	104.7 (4)
C9—C8—H8B	108.9	C55—C54—C53	111.5 (4)
C7—C8—H8B	108.9	C60—C54—C53	110.4 (4)
H8A—C8—H8B	107.7	C62—C54—C53	106.9 (4)
C8—C9—C10	111.3 (5)	C54—C55—C56	114.3 (4)
C8—C9—H9A	109.4	C54—C55—H55A	108.7
C10—C9—H9A	109.4	C56—C55—H55A	108.7
C8—C9—H9B	109.4	C54—C55—H55B	108.7
C10—C9—H9B	109.4	C56—C55—H55B	108.7
H9A—C9—H9B	108.0	H55A—C55—H55B	107.6
C5—C10—C9	112.2 (5)	C55—C56—C51	110.9 (4)
C5—C10—H10A	109.2	C55—C56—H56A	109.5
C9—C10—H10A	109.2	C51—C56—H56A	109.5
C5—C10—H10B	109.2	C55—C56—H56B	109.5
C9—C10—H10B	109.2	C51—C56—H56B	109.5
H10A—C10—H10B	107.9	H56A—C56—H56B	108.0
C12—C11—C4	108.7 (4)	C58—C57—C53	111.9 (5)
C12—C11—H11A	109.9	C58—C57—H57A	109.2
C4—C11—H11A	109.9	C53—C57—H57A	109.2
C12—C11—H11B	109.9	C58—C57—H57B	109.2
C4—C11—H11B	109.9	C53—C57—H57B	109.2
H11A—C11—H11B	108.3	H57A—C57—H57B	107.9
C15—C12—C13	108.5 (5)	C61—C58—C57	108.7 (6)
C15—C12—C11	109.1 (6)	C61—C58—C59	105.7 (6)
C13—C12—C11	109.2 (6)	C57—C58—C59	109.7 (6)
C15—C12—H12	110.0	C61—C58—H58	110.9
C13—C12—H12	110.0	C57—C58—H58	110.9
C11—C12—H12	110.0	C59—C58—H58	110.9
C14—C13—C21	127.4 (8)	C60—C59—C67	127.5 (8)
C14—C13—C12	111.6 (6)	C60—C59—C58	113.7 (6)
C21—C13—C12	121.0 (7)	C67—C59—C58	118.8 (7)
C13—C14—C3	116.7 (6)	C59—C60—C54	113.2 (6)
C13—C14—H14	121.6	C59—C60—H60	123.4
C3—C14—H14	121.6	C54—C60—H60	123.4
C12—C15—C16	110.2 (5)	C58—C61—C62	110.9 (6)
C12—C15—H15A	109.6	C58—C61—H61A	109.5
C16—C15—H15A	109.6	C62—C61—H61A	109.5
C12—C15—H15B	109.6	C58—C61—H61B	109.5
C16—C15—H15B	109.6	C62—C61—H61B	109.5
H15A—C15—H15B	108.1	H61A—C61—H61B	108.0
C15—C16—C20	111.0 (4)	C66—C62—C54	113.8 (5)
C15—C16—C3	109.2 (4)	C66—C62—C61	111.8 (5)
C20—C16—C3	111.3 (4)	C54—C62—C61	109.4 (5)
C15—C16—H16	108.4	C66—C62—H62	107.2
C20—C16—H16	108.4	C54—C62—H62	107.2
C3—C16—H16	108.4	C61—C62—H62	107.2
C5—C17—H17A	109.5	O5—C63—C50	114.4 (5)
C5—C17—H17B	109.5	O5—C63—H63A	108.7
H17A—C17—H17B	109.5	C50—C63—H63A	108.7
C5—C17—H17C	109.5	O5—C63—H63B	108.7
H17A—C17—H17C	109.5	C50—C63—H63B	108.7
H17B—C17—H17C	109.5	H63A—C63—H63B	107.6
C7—C18—H18A	109.5	C50—C64—H64A	109.5
C7—C18—H18B	109.5	C50—C64—H64B	109.5
H18A—C18—H18B	109.5	H64A—C64—H64B	109.5
C7—C18—H18C	109.5	C50—C64—H64C	109.5
H18A—C18—H18C	109.5	H64A—C64—H64C	109.5
H18B—C18—H18C	109.5	H64B—C64—H64C	109.5
O1—C19—C7	114.9 (5)	C52—C65—H65A	109.5
O1—C19—H19A	108.5	C52—C65—H65B	109.5
C7—C19—H19A	108.5	H65A—C65—H65B	109.5
O1—C19—H19B	108.5	C52—C65—H65C	109.5
C7—C19—H19B	108.5	H65A—C65—H65C	109.5
H19A—C19—H19B	107.5	H65B—C65—H65C	109.5
O2—C20—C16	111.9 (4)	O6—C66—C62	114.5 (5)
O2—C20—H20A	109.2	O6—C66—H66A	108.6
C16—C20—H20A	109.2	C62—C66—H66A	108.6
O2—C20—H20B	109.2	O6—C66—H66B	108.6
C16—C20—H20B	109.2	C62—C66—H66B	108.6
H20A—C20—H20B	107.9	H66A—C66—H66B	107.6
C23—C21—C13	115.7 (8)	C68—C67—C69	115.0 (8)
C23—C21—C22	92.7 (8)	C68—C67—C59	118.3 (7)
C13—C21—C22	107.2 (8)	C69—C67—C59	115.9 (6)
C23—C21—H21	113.1	C68—C67—H67	101.1
C13—C21—H21	113.1	C69—C67—H67	101.1
C22—C21—H21	113.1	C59—C67—H67	101.1
C21—C22—H22A	109.5	C67—C68—H68A	109.5
C21—C22—H22B	109.5	C67—C68—H68B	109.5
H22A—C22—H22B	109.5	H68A—C68—H68B	109.5
C21—C22—H22C	109.5	C67—C68—H68C	109.5
H22A—C22—H22C	109.5	H68A—C68—H68C	109.5
H22B—C22—H22C	109.5	H68B—C68—H68C	109.5
C21—C23—H23A	109.5	C67—C69—H69A	109.5
C21—C23—H23B	109.5	C67—C69—H69B	109.5
H23A—C23—H23B	109.5	H69A—C69—H69B	109.5
C21—C23—H23C	109.5	C67—C69—H69C	109.5
H23A—C23—H23C	109.5	H69A—C69—H69C	109.5
H23B—C23—H23C	109.5	H69B—C69—H69C	109.5
C42—O3—H3O	109.5	C86—O7—H7O	109.5
C43—O4—H4O	109.5	C89—O8—H8O	109.4
C25—C24—C29	113.1 (5)	C71—C70—C75	113.3 (5)
C25—C24—H24A	109.0	C71—C70—H70A	108.9
C29—C24—H24A	109.0	C75—C70—H70A	108.9
C25—C24—H24B	109.0	C71—C70—H70B	108.9
C29—C24—H24B	109.0	C75—C70—H70B	108.9
H24A—C24—H24B	107.8	H70A—C70—H70B	107.7
C26—C25—C24	109.9 (5)	C72—C71—C70	110.4 (5)
C26—C25—H25A	109.7	C72—C71—H71A	109.6
C24—C25—H25A	109.7	C70—C71—H71A	109.6
C26—C25—H25B	109.7	C72—C71—H71B	109.6
C24—C25—H25B	109.7	C70—C71—H71B	109.6
H25A—C25—H25B	108.2	H71A—C71—H71B	108.1
C25—C26—C27	113.9 (5)	C71—C72—C73	113.6 (5)
C25—C26—H26A	108.8	C71—C72—H72A	108.9
C27—C26—H26A	108.8	C73—C72—H72A	108.9
C25—C26—H26B	108.8	C71—C72—H72B	108.9
C27—C26—H26B	108.8	C73—C72—H72B	108.9
H26A—C26—H26B	107.7	H72A—C72—H72B	107.7
C26—C27—C41	112.3 (5)	C86—C73—C72	109.3 (5)
C26—C27—C42	108.4 (4)	C86—C73—C74	110.7 (4)
C41—C27—C42	104.4 (5)	C72—C73—C74	109.2 (4)
C26—C27—C28	108.1 (4)	C86—C73—C87	102.6 (5)
C41—C27—C28	114.5 (4)	C72—C73—C87	111.9 (5)
C42—C27—C28	109.0 (4)	C74—C73—C87	113.1 (5)
C30—C28—C27	115.4 (4)	C76—C74—C73	116.0 (4)
C30—C28—C29	110.4 (4)	C76—C74—C75	109.9 (4)
C27—C28—C29	116.4 (4)	C73—C74—C75	118.0 (4)
C30—C28—H28	104.3	C76—C74—H74	103.6
C27—C28—H28	104.3	C73—C74—H74	103.6
C29—C28—H28	104.3	C75—C74—H74	103.6
C24—C29—C40	108.5 (4)	C70—C75—C79	106.5 (4)
C24—C29—C28	107.9 (4)	C70—C75—C88	109.2 (4)
C40—C29—C28	112.8 (4)	C79—C75—C88	112.0 (4)
C24—C29—C33	107.8 (4)	C70—C75—C74	108.0 (4)
C40—C29—C33	111.9 (4)	C79—C75—C74	109.1 (4)
C28—C29—C33	107.7 (4)	C88—C75—C74	111.8 (4)
C28—C30—C31	110.9 (4)	C74—C76—C77	110.9 (4)
C28—C30—H30A	109.5	C74—C76—H76A	109.5
C31—C30—H30A	109.5	C77—C76—H76A	109.5
C28—C30—H30B	109.5	C74—C76—H76B	109.5
C31—C30—H30B	109.5	C77—C76—H76B	109.5
H30A—C30—H30B	108.1	H76A—C76—H76B	108.0
C32—C31—C30	114.4 (4)	C78—C77—C76	113.8 (4)
C32—C31—H31A	108.7	C78—C77—H77A	108.8
C30—C31—H31A	108.7	C76—C77—H77A	108.8
C32—C31—H31B	108.7	C78—C77—H77B	108.8
C30—C31—H31B	108.7	C76—C77—H77B	108.8
H31A—C31—H31B	107.6	H77A—C77—H77B	107.7
C39—C32—C31	114.2 (4)	C84—C78—C77	114.9 (5)
C39—C32—C33	110.1 (4)	C84—C78—C83	107.0 (4)
C31—C32—C33	110.3 (4)	C77—C78—C83	110.7 (4)
C39—C32—C37	105.3 (4)	C84—C78—C79	108.6 (4)
C31—C32—C37	110.8 (4)	C77—C78—C79	110.2 (4)
C33—C32—C37	105.7 (4)	C83—C78—C79	104.9 (4)
C34—C33—C32	108.7 (4)	C80—C79—C75	115.1 (4)
C34—C33—C29	113.3 (4)	C80—C79—C78	107.6 (4)
C32—C33—C29	115.9 (4)	C75—C79—C78	115.0 (4)
C34—C33—H33	106.1	C80—C79—H79	106.2
C32—C33—H33	106.1	C75—C79—H79	106.2
C29—C33—H33	106.1	C78—C79—H79	106.2
C33—C34—C35	109.3 (5)	C81—C80—C79	111.6 (4)
C33—C34—H34A	109.8	C81—C80—H80A	109.3
C35—C34—H34A	109.8	C79—C80—H80A	109.3
C33—C34—H34B	109.8	C81—C80—H80B	109.3
C35—C34—H34B	109.8	C79—C80—H80B	109.3
H34A—C34—H34B	108.3	H80A—C80—H80B	108.0
C38—C35—C36	110.1 (5)	C85—C81—C82	108.7 (5)
C38—C35—C34	109.1 (5)	C85—C81—C80	108.4 (5)
C36—C35—C34	106.6 (5)	C82—C81—C80	108.8 (6)
C38—C35—H35	110.3	C85—C81—H81A	110.3
C36—C35—H35	110.3	C82—C81—H81A	110.3
C34—C35—H35	110.3	C80—C81—H81A	110.3
C37—C36—C35	109.4 (5)	C81—C82—C83	109.1 (5)
C37—C36—H36A	109.8	C81—C82—H82A	109.9
C35—C36—H36A	109.8	C83—C82—H82A	109.9
C37—C36—H36B	109.8	C81—C82—H82B	109.9
C35—C36—H36B	109.8	C83—C82—H82B	109.9
H36A—C36—H36B	108.3	H82A—C82—H82B	108.3
C43—C37—C36	113.1 (4)	C89—C83—C78	111.2 (4)
C43—C37—C32	110.7 (4)	C89—C83—C82	110.3 (5)
C36—C37—C32	109.8 (4)	C78—C83—C82	109.4 (5)
C43—C37—H37	107.7	C89—C83—H83A	108.6
C36—C37—H37	107.7	C78—C83—H83A	108.6
C32—C37—H37	107.7	C82—C83—H83A	108.6
C39—C38—C35	111.6 (5)	C85—C84—C78	116.3 (5)
C39—C38—C44	122.7 (7)	C85—C84—H84	121.8
C35—C38—C44	125.4 (7)	C78—C84—H84	121.8
C38—C39—C32	117.2 (6)	C84—C85—C81	112.2 (5)
C38—C39—H39	121.4	C84—C85—C90	127.6 (7)
C32—C39—H39	121.4	C81—C85—C90	120.1 (6)
C29—C40—H40A	109.5	O7—C86—C73	115.9 (5)
C29—C40—H40B	109.5	O7—C86—H86A	108.3
H40A—C40—H40B	109.5	C73—C86—H86A	108.3
C29—C40—H40C	109.5	O7—C86—H86B	108.3
H40A—C40—H40C	109.5	C73—C86—H86B	108.3
H40B—C40—H40C	109.5	H86A—C86—H86B	107.4
C27—C41—H41A	109.5	C73—C87—H87A	109.5
C27—C41—H41B	109.5	C73—C87—H87B	109.5
H41A—C41—H41B	109.5	H87A—C87—H87B	109.5
C27—C41—H41C	109.5	C73—C87—H87C	109.5
H41A—C41—H41C	109.5	H87A—C87—H87C	109.5
H41B—C41—H41C	109.5	H87B—C87—H87C	109.5
O3—C42—C27	113.8 (5)	C75—C88—H88A	109.5
O3—C42—H42A	108.8	C75—C88—H88B	109.5
C27—C42—H42A	108.8	H88A—C88—H88B	109.5
O3—C42—H42B	108.8	C75—C88—H88C	109.5
C27—C42—H42B	108.8	H88A—C88—H88C	109.5
H42A—C42—H42B	107.7	H88B—C88—H88C	109.5
O4—C43—C37	111.2 (4)	O8—C89—C83	111.6 (5)
O4—C43—H43A	109.4	O8—C89—H89A	109.3
C37—C43—H43A	109.4	C83—C89—H89A	109.3
O4—C43—H43B	109.4	O8—C89—H89B	109.3
C37—C43—H43B	109.4	C83—C89—H89B	109.3
H43A—C43—H43B	108.0	H89A—C89—H89B	108.0
C46—C44—C45	114.3 (7)	C91—C90—C85	116.7 (7)
C46—C44—C38	115.4 (6)	C91—C90—C92	112.3 (7)
C45—C44—C38	113.7 (6)	C85—C90—C92	113.8 (6)
C46—C44—H44A	103.9	C91—C90—H90	104.1
C45—C44—H44A	103.9	C85—C90—H90	104.1
C38—C44—H44A	103.9	C92—C90—H90	104.1
C44—C45—H45A	109.5	C90—C91—H91A	109.5
C44—C45—H45B	109.5	C90—C91—H91B	109.5
H45A—C45—H45B	109.5	H91A—C91—H91B	109.5
C44—C45—H45C	109.5	C90—C91—H91C	109.5
H45A—C45—H45C	109.5	H91A—C91—H91C	109.5
H45B—C45—H45C	109.5	H91B—C91—H91C	109.5
C44—C46—H46A	109.5	C90—C92—H92A	109.5
C44—C46—H46B	109.5	C90—C92—H92B	109.5
H46A—C46—H46B	109.5	H92A—C92—H92B	109.5
C44—C46—H46C	109.5	C90—C92—H92C	109.5
H46A—C46—H46C	109.5	H92A—C92—H92C	109.5
H46B—C46—H46C	109.5	H92B—C92—H92C	109.5
			
C6—C1—C2—C3	53.8 (6)	C52—C47—C48—C49	−58.0 (8)
C1—C2—C3—C14	78.9 (5)	C47—C48—C49—C50	57.5 (8)
C1—C2—C3—C4	−44.0 (6)	C48—C49—C50—C64	74.2 (7)
C1—C2—C3—C16	−161.8 (4)	C48—C49—C50—C51	−53.3 (7)
C14—C3—C4—C11	51.3 (6)	C48—C49—C50—C63	−171.8 (5)
C2—C3—C4—C11	176.4 (5)	C49—C50—C51—C56	−179.1 (5)
C16—C3—C4—C11	−62.4 (5)	C64—C50—C51—C56	55.2 (6)
C14—C3—C4—C5	−79.7 (5)	C63—C50—C51—C56	−60.9 (6)
C2—C3—C4—C5	45.4 (6)	C49—C50—C51—C52	50.8 (6)
C16—C3—C4—C5	166.6 (4)	C64—C50—C51—C52	−74.9 (6)
C3—C4—C5—C10	−169.4 (5)	C63—C50—C51—C52	169.0 (5)
C11—C4—C5—C10	61.7 (6)	C48—C47—C52—C51	52.9 (7)
C3—C4—C5—C17	71.6 (6)	C48—C47—C52—C65	−70.2 (7)
C11—C4—C5—C17	−57.3 (6)	C48—C47—C52—C53	168.5 (5)
C3—C4—C5—C6	−52.3 (6)	C50—C51—C52—C47	−50.6 (6)
C11—C4—C5—C6	178.9 (5)	C56—C51—C52—C47	177.1 (5)
C2—C1—C6—C5	−62.5 (5)	C50—C51—C52—C65	69.3 (6)
C2—C1—C6—C7	163.7 (4)	C56—C51—C52—C65	−63.0 (6)
C10—C5—C6—C1	176.3 (5)	C50—C51—C52—C53	−167.2 (4)
C17—C5—C6—C1	−62.9 (6)	C56—C51—C52—C53	60.5 (5)
C4—C5—C6—C1	59.4 (5)	C47—C52—C53—C57	63.3 (6)
C10—C5—C6—C7	−50.5 (6)	C51—C52—C53—C57	179.1 (5)
C17—C5—C6—C7	70.3 (6)	C65—C52—C53—C57	−56.0 (6)
C4—C5—C6—C7	−167.4 (4)	C47—C52—C53—C54	−170.1 (5)
C1—C6—C7—C19	−60.9 (6)	C51—C52—C53—C54	−54.3 (6)
C5—C6—C7—C19	167.7 (5)	C65—C52—C53—C54	70.6 (6)
C1—C6—C7—C18	57.1 (6)	C57—C53—C54—C55	177.0 (5)
C5—C6—C7—C18	−74.3 (6)	C52—C53—C54—C55	46.8 (6)
C1—C6—C7—C8	179.4 (4)	C57—C53—C54—C60	51.6 (6)
C5—C6—C7—C8	48.0 (6)	C52—C53—C54—C60	−78.6 (6)
C19—C7—C8—C9	−170.9 (5)	C57—C53—C54—C62	−61.8 (5)
C18—C7—C8—C9	73.8 (6)	C52—C53—C54—C62	168.0 (4)
C6—C7—C8—C9	−50.7 (7)	C60—C54—C55—C56	78.9 (5)
C7—C8—C9—C10	56.9 (8)	C62—C54—C55—C56	−164.5 (4)
C17—C5—C10—C9	−70.0 (6)	C53—C54—C55—C56	−45.6 (6)
C6—C5—C10—C9	54.1 (6)	C54—C55—C56—C51	54.5 (6)
C4—C5—C10—C9	169.2 (5)	C50—C51—C56—C55	164.1 (4)
C8—C9—C10—C5	−59.2 (7)	C52—C51—C56—C55	−62.2 (6)
C3—C4—C11—C12	3.1 (7)	C54—C53—C57—C58	3.9 (7)
C5—C4—C11—C12	135.7 (5)	C52—C53—C57—C58	134.3 (6)
C4—C11—C12—C15	60.1 (7)	C53—C57—C58—C61	57.9 (8)
C4—C11—C12—C13	−58.3 (7)	C53—C57—C58—C59	−57.3 (7)
C15—C12—C13—C14	−59.4 (7)	C61—C58—C59—C60	−61.3 (7)
C11—C12—C13—C14	59.4 (7)	C57—C58—C59—C60	55.8 (7)
C15—C12—C13—C21	120.3 (7)	C61—C58—C59—C67	118.2 (6)
C11—C12—C13—C21	−120.9 (7)	C57—C58—C59—C67	−124.8 (6)
C21—C13—C14—C3	−179.8 (6)	C67—C59—C60—C54	−177.6 (6)
C12—C13—C14—C3	−0.2 (8)	C58—C59—C60—C54	1.8 (7)
C2—C3—C14—C13	179.2 (5)	C55—C54—C60—C59	178.0 (5)
C4—C3—C14—C13	−56.4 (6)	C62—C54—C60—C59	57.8 (6)
C16—C3—C14—C13	56.9 (6)	C53—C54—C60—C59	−57.0 (6)
C13—C12—C15—C16	57.1 (7)	C57—C58—C61—C62	−60.8 (8)
C11—C12—C15—C16	−61.7 (7)	C59—C58—C61—C62	56.9 (7)
C12—C15—C16—C20	−123.7 (6)	C55—C54—C62—C66	−53.2 (6)
C12—C15—C16—C3	−0.6 (7)	C60—C54—C62—C66	67.9 (6)
C14—C3—C16—C15	−53.7 (5)	C53—C54—C62—C66	−174.9 (4)
C2—C3—C16—C15	−176.9 (4)	C55—C54—C62—C61	−179.0 (5)
C4—C3—C16—C15	61.7 (5)	C60—C54—C62—C61	−57.9 (6)
C14—C3—C16—C20	69.2 (5)	C53—C54—C62—C61	59.3 (6)
C2—C3—C16—C20	−54.0 (6)	C58—C61—C62—C66	−125.4 (6)
C4—C3—C16—C20	−175.3 (4)	C58—C61—C62—C54	1.5 (8)
C18—C7—C19—O1	−176.5 (5)	C49—C50—C63—O5	65.7 (6)
C8—C7—C19—O1	65.9 (6)	C64—C50—C63—O5	−175.3 (5)
C6—C7—C19—O1	−54.2 (7)	C51—C50—C63—O5	−52.4 (7)
C15—C16—C20—O2	−54.4 (6)	C54—C62—C66—O6	−173.7 (4)
C3—C16—C20—O2	−176.3 (4)	C61—C62—C66—O6	−49.1 (7)
C14—C13—C21—C23	94.8 (11)	C60—C59—C67—C68	38.7 (11)
C12—C13—C21—C23	−84.9 (10)	C58—C59—C67—C68	−140.7 (8)
C14—C13—C21—C22	−7.0 (11)	C60—C59—C67—C69	−104.0 (9)
C12—C13—C21—C22	173.4 (7)	C58—C59—C67—C69	76.6 (10)
C29—C24—C25—C26	−58.6 (7)	C75—C70—C71—C72	−58.5 (7)
C24—C25—C26—C27	59.1 (7)	C70—C71—C72—C73	57.4 (7)
C25—C26—C27—C41	73.3 (6)	C71—C72—C73—C86	−172.2 (5)
C25—C26—C27—C42	−171.9 (5)	C71—C72—C73—C74	−51.0 (7)
C25—C26—C27—C28	−54.0 (6)	C71—C72—C73—C87	74.9 (7)
C26—C27—C28—C30	−177.1 (4)	C86—C73—C74—C76	−57.7 (6)
C41—C27—C28—C30	56.9 (6)	C72—C73—C74—C76	−178.0 (5)
C42—C27—C28—C30	−59.5 (5)	C87—C73—C74—C76	56.7 (7)
C26—C27—C28—C29	51.0 (6)	C86—C73—C74—C75	168.8 (5)
C41—C27—C28—C29	−75.0 (6)	C72—C73—C74—C75	48.4 (6)
C42—C27—C28—C29	168.6 (4)	C87—C73—C74—C75	−76.8 (6)
C25—C24—C29—C40	−69.1 (6)	C71—C70—C75—C79	169.6 (5)
C25—C24—C29—C28	53.4 (6)	C71—C70—C75—C88	−69.3 (6)
C25—C24—C29—C33	169.5 (4)	C71—C70—C75—C74	52.5 (6)
C30—C28—C29—C24	174.9 (4)	C76—C74—C75—C70	174.9 (5)
C27—C28—C29—C24	−50.9 (5)	C73—C74—C75—C70	−49.0 (6)
C30—C28—C29—C40	−65.3 (5)	C76—C74—C75—C79	59.4 (5)
C27—C28—C29—C40	68.9 (5)	C73—C74—C75—C79	−164.5 (4)
C30—C28—C29—C33	58.8 (5)	C76—C74—C75—C88	−64.9 (5)
C27—C28—C29—C33	−167.1 (4)	C73—C74—C75—C88	71.2 (6)
C27—C28—C30—C31	164.4 (4)	C73—C74—C76—C77	160.6 (4)
C29—C28—C30—C31	−60.9 (5)	C75—C74—C76—C77	−62.3 (6)
C28—C30—C31—C32	55.6 (6)	C74—C76—C77—C78	57.4 (6)
C30—C31—C32—C39	77.3 (5)	C76—C77—C78—C84	75.7 (6)
C30—C31—C32—C33	−47.3 (5)	C76—C77—C78—C83	−163.0 (4)
C30—C31—C32—C37	−164.0 (4)	C76—C77—C78—C79	−47.4 (6)
C39—C32—C33—C34	49.8 (6)	C70—C75—C79—C80	65.6 (6)
C31—C32—C33—C34	176.7 (4)	C88—C75—C79—C80	−53.8 (6)
C37—C32—C33—C34	−63.5 (5)	C74—C75—C79—C80	−178.1 (4)
C39—C32—C33—C29	−79.1 (5)	C70—C75—C79—C78	−168.6 (4)
C31—C32—C33—C29	47.8 (5)	C88—C75—C79—C78	72.0 (5)
C37—C32—C33—C29	167.6 (4)	C74—C75—C79—C78	−52.3 (5)
C24—C29—C33—C34	63.8 (6)	C84—C78—C79—C80	49.3 (6)
C40—C29—C33—C34	−55.5 (6)	C77—C78—C79—C80	175.9 (5)
C28—C29—C33—C34	180.0 (4)	C83—C78—C79—C80	−64.9 (5)
C24—C29—C33—C32	−169.6 (4)	C84—C78—C79—C75	−80.3 (5)
C40—C29—C33—C32	71.1 (5)	C77—C78—C79—C75	46.3 (6)
C28—C29—C33—C32	−53.5 (5)	C83—C78—C79—C75	165.6 (4)
C32—C33—C34—C35	3.3 (6)	C75—C79—C80—C81	135.3 (5)
C29—C33—C34—C35	133.6 (5)	C78—C79—C80—C81	5.8 (7)
C33—C34—C35—C38	−58.3 (6)	C79—C80—C81—C85	−59.9 (7)
C33—C34—C35—C36	60.5 (7)	C79—C80—C81—C82	58.2 (7)
C38—C35—C36—C37	54.0 (6)	C85—C81—C82—C83	56.9 (6)
C34—C35—C36—C37	−64.2 (7)	C80—C81—C82—C83	−60.9 (7)
C35—C36—C37—C43	−121.4 (5)	C84—C78—C83—C89	68.6 (6)
C35—C36—C37—C32	2.8 (6)	C77—C78—C83—C89	−57.3 (6)
C39—C32—C37—C43	69.6 (5)	C79—C78—C83—C89	−176.1 (4)
C31—C32—C37—C43	−54.4 (5)	C84—C78—C83—C82	−53.5 (6)
C33—C32—C37—C43	−173.8 (4)	C77—C78—C83—C82	−179.4 (4)
C39—C32—C37—C36	−55.9 (5)	C79—C78—C83—C82	61.8 (5)
C31—C32—C37—C36	−179.9 (4)	C81—C82—C83—C89	−122.6 (6)
C33—C32—C37—C36	60.6 (5)	C81—C82—C83—C78	0.0 (7)
C36—C35—C38—C39	−56.5 (7)	C77—C78—C84—C85	178.9 (5)
C34—C35—C38—C39	60.1 (7)	C83—C78—C84—C85	55.5 (6)
C36—C35—C38—C44	117.5 (6)	C79—C78—C84—C85	−57.3 (6)
C34—C35—C38—C44	−125.9 (6)	C78—C84—C85—C81	2.1 (8)
C35—C38—C39—C32	−2.7 (7)	C78—C84—C85—C90	−176.1 (6)
C44—C38—C39—C32	−176.9 (5)	C82—C81—C85—C84	−61.0 (7)
C31—C32—C39—C38	−178.8 (5)	C80—C81—C85—C84	57.1 (7)
C33—C32—C39—C38	−54.0 (6)	C82—C81—C85—C90	117.4 (6)
C37—C32—C39—C38	59.4 (6)	C80—C81—C85—C90	−124.5 (6)
C26—C27—C42—O3	63.9 (6)	C72—C73—C86—O7	63.6 (6)
C41—C27—C42—O3	−176.2 (5)	C74—C73—C86—O7	−56.6 (6)
C28—C27—C42—O3	−53.5 (6)	C87—C73—C86—O7	−177.6 (5)
C36—C37—C43—O4	−51.1 (6)	C78—C83—C89—O8	−175.5 (4)
C32—C37—C43—O4	−174.8 (4)	C82—C83—C89—O8	−53.9 (6)
C39—C38—C44—C46	−116.5 (9)	C84—C85—C90—C91	34.4 (11)
C35—C38—C44—C46	70.2 (9)	C81—C85—C90—C91	−143.6 (8)
C39—C38—C44—C45	108.7 (8)	C84—C85—C90—C92	−98.9 (9)
C35—C38—C44—C45	−64.6 (10)	C81—C85—C90—C92	83.1 (9)

### Hydrogen-bond geometry (Å, °)

**Table:**

*D*—H···*A*	*D*—H	H···*A*	*D*···*A*	*D*—H···*A*
O1—H1O···O6^i^	0.82	1.99	2.717 (6)	148
O2—H2O···O7^ii^	0.85	1.84	2.692 (5)	179
O3—H3O···O2^iii^	0.82	1.92	2.712 (5)	163
O4—H4O···O5^iv^	0.82	2.11	2.694 (5)	128
O5—H5O···O8^v^	0.82	1.92	2.682 (5)	155
O6—H6O···O3^vi^	0.82	2.03	2.704 (6)	139
O7—H7O···O4^vii^	0.82	2.02	2.692 (5)	139
O8—H8O···O1^viii^	0.82	1.87	2.677 (6)	166

Symmetry codes: (i) *x*−1, *y*, *z*−1; (ii) −*x*+1, *y*+1/2, −*z*; (iii) −*x*+1, *y*−1/2, −*z*+1; (iv) −*x*, *y*−1/2, −*z*+2; (v) −*x*, *y*+1/2, −*z*+1; (vi) −*x*+1, *y*+1/2, −*z*+2; (vii) *x*+1, *y*, *z*−1; (viii) −*x*, *y*−1/2, −*z*.

## References

[bb1] Aldrich, P. H. (1971). US Patent No. 3 562 243.

[bb2] Enraf–Nonius (1994). *CAD-4 EXPRESS* Enraf–Nonius, Delft, The Netherlands.

[bb3] Harms, K. & Wocadlo, S. (1995). *XCAD4* University of Marburg, Germany.

[bb4] North, A. C. T., Phillips, D. C. & Mathews, F. S. (1968). *Acta Cryst.* A**24**, 351–359.

[bb5] Sheldrick, G. M. (2008). *Acta Cryst.* A**64**, 112–122. 10.1107/S0108767307043930 18156677

[bb6] Wang, H.-X., Shang, S.-B., Yin, Y.-B., Rao, X.-P. & Xu, X. (2009). *Acta Cryst.* E**65**, o1521. 10.1107/S1600536809021059 PMC2969219 21582812

